# Decision support software-guided medication reviews in elderly patients with polypharmacy: a prospective analysis of routine data from community pharmacies (OPtiMed study protocol)

**DOI:** 10.1186/s40545-022-00495-z

**Published:** 2022-12-09

**Authors:** Stefan Maierhöfer, Isabell Waltering, Mareike Jacobs, Gudrun Würthwein, Meike Appelrath, Susanne Koling, Georg Hempel

**Affiliations:** 1 Department of Pharmaceutical and Medicinal Chemistry — Clinical Pharmacy, Westfaelische Wilhelms-University, Muenster, Germany; 2 Viandar GmbH, Lengerich, Germany; 3 Clinic for Pediatrics and Adolescent Medicine — Evangelical Hospital Hamm, Hamm, Germany

**Keywords:** Polypharmacy, Pharmaceutical care, Medication safety, Medication review, Clinical decision support system, Health information technology

## Abstract

**Background:**

Pharmacist-led medication reviews are considered a valuable measure to address risks of polypharmacy. The software Medinspector^®^ is used in community pharmacies to assist the performance of this complex service by structuring the medication review process and supporting pharmacists in their decision-making with targeted clinical knowledge. Key feature is a computerized risk assessment of both the initial and adjusted medication regimen of a patient in multiple domains, thus aiming to support the identification and solving of drug-related problems. This study will examine the effects of medication reviews performed with the clinical decision support system in daily routine practice on medication-related and patient-reported outcomes in elderly patients with polypharmacy.

**Methods:**

A prospective, before–after observational study is conducted in German community pharmacies aiming to include 148 patients aged 65 or older, who chronically use five or more active pharmaceutical substances with systemic effects and utilize the software-supported medication review service. The study is based on routine documentation within the software over the course of the medication review, including a patient’s baseline medication, the medication proposed by pharmacists, and the final medication regimen. A software-implemented questionnaire comprising self-developed and literature-derived instruments is used to collect patient-reported outcome data at baseline and follow-up. Primary outcome is the appropriateness of medication measured with an adapted version of the Medication Appropriateness Index (MAI). Secondary medication-related outcomes are medication underuse, exposition towards anticholinergic/sedative drugs, number of drugs in long-term use and the implementation of pharmacist-proposed medication adjustments by the physicians. Secondary patient-reported outcomes are symptom burden, medication-related quality of life, adherence, fulfillment of medication review-related goals, and perception of the service.

**Discussion:**

With the recently introduced remuneration of community pharmacist-led MR in Germany, the demand for digital tools supporting the MR process is assumed to rise. The OPtiMed-study is expected to create evidence on the effects of a novel tool on patient care in a vulnerable patient population.

*Trial registration* German Clinical Trials Register, DRKS00027410. Registered 22 December 2021, https://www.drks.de/drks_web/navigate.do?navigationId=trial.HTML&TRIAL_ID=DRKS00027410. Also available on the WHO meta-registry: https://trialsearch.who.int/?TrialID=DRKS00027410

## Background

### Polypharmacy

Assuring patient safety in polypharmacy has been identified as one of the greatest public health challenges of our time [1, 2]. Although, no uniform definition exists, polypharmacy is generally understood as the simultaneous use of five or more drugs [3, 4]. Irrespective of its definition, polypharmacy in older adults is widespread and growing [5]. A recent European analysis revealed that almost one-third of community-dwelling elderly (≥ 65 years) used five or more drugs per day [6]. And essentially, polypharmacy increases the risk of various adverse consequences, including inappropriate medication use, underuse of indicated drugs, poor adherence, adverse drug reactions, reduced quality of life, morbidity and even mortality [3, 6–9]. One approach of addressing the risks of polypharmacy are medication reviews (MR) conducted by pharmacists.

### Medication review in polypharmacy

Medication review is a structured in depth assessment of a patient’s medication with the purpose of enhancing appropriateness of medication use, thus preventing harm and improving patient outcomes [10].

Today, MR are considered a valuable instrument for managing polypharmacy even though robust evidence from systematic reviews and meta-analysis demonstrating consistent effects on clinical outcomes and quality of life are lacking and their impact on medication appropriateness remains unclear according to a recent Cochrane Review [11, 12].

From pharmacist’s perspective, performing MR is a challenging task. Involvement of both patient and prescribing physician requires a highly structured process to be equally practical and efficient [13]. Furthermore, tailoring a drug regimen to patient’s needs involves complex decision-making with consideration of large amounts of data including multiple drugs, comorbidities and individual preferences [14]. Hence, there is a demand for tools that structure and standardize the MR process, enhance coordination between involved parties and support healthcare professionals with decision-making on choosing the optimal medication based on current evidence. The use of health information technology, such as clinical decision support systems (CDSS), is a promising strategy in this regard and is promoted by public policy in several countries [15, 16].

### Clinical decision support systems to optimize medication use

Clinical decision support systems are software programs designed to support healthcare professionals in daily decision-making by providing patient-specific information, e.g., alerts for inappropriate drug dosing regarding a patient’s renal function [17]. An overview reports evidence of CDSS use for a reduction in medication errors, but their effect on clinical outcomes is inconclusive. However, interventions and embedded CDSS, type and qualification of software users as well as healthcare systems varies substantially among studies. Hence, the transferability of results is questionable [18]. In the ambulatory setting, only few studies investigated effects of CDSS use for structured MR considering all drugs used and multiple types of drug-related problems (DRP) [19, 20].

### The medication review software Medinspector^®^

The software utilized in this project (Medinspector^®^, Viandar GmbH, Lengerich, Germany) was developed to support the execution of MR in community pharmacies located in Germany [21]. Although all types of MR can be performed with the software, it is specifically designed to assist the conduction of intermediate and advanced MR (Type 2a/b and 3 according to the Pharmaceutical Care Network Europe (PCNE) definition) with clinical and/or patient information being available [10]. The web-based tool guides pharmacists and pharmacist-technicians stepwise through the MR process: from collection of patient information to a decision supported evaluation and management of medication therapy up to a follow-up survey for a de novo assessment of patient needs after implementation of therapy modifications. Typically required documents for MR are software-implemented or automatically generated based on recorded data throughout the process, e.g., (i) a document to inform the prescribing physician about identified DRP and corresponding intervention proposals or (ii) the German Federal Medication Plan to be handed out to the patient. Key software feature is a clinical decision support functionality comprising a risk-prioritized presentation of DRP in various domains based on individual patient data and knowledge from pharmaceutical databases. In case of uncertainty regarding the relevance and management of DRP, community pharmacists can request case-specific support from pharmacist-experts trained in MR. An associated training concept includes live-online and web-based trainings, video-tutorials, and exemplary patient cases.

In Germany, to the best of our knowledge, an evaluation of a CDSS utilized to assist the conduction of ambulatory MR has not been published yet. Hence, research in this area is desired.

## Design

### Aim

The aim of the OPtiMed study (Optimizing Polypharmacotherapy in Geriatric Patients with the Medication Review Software Medinspector^®^) is to evaluate the effects of routinely performed, software-supported MR on medication-related and patient-reported outcomes in geriatric community pharmacy patients with polypharmacy.

### Study design

This study is designed as a single-arm, multicenter, prospective before–after observational study.

### Participating pharmacies

We will analyze routine data collected in German community pharmacies using the Medinspector^®^ software to perform MR. The software is nationwide available as subscription-based pricing model. In addition, the software is provided for free to community pharmacies participating in the MR training program “ATHINA” (formerly “Apo-AMTS”) in the Pharmacists’ Chamber of Westphalia-Lippe, a region in Germany [22]. Within the program, pharmacists/pre-registration students supervised by pharmacists (in the text reported as “pharmacists”) have to perform four to five intermediate MRs Type 2a which can be executed with the software.

Software-users are pharmacists and pharmacist technicians. Varying authorizations within the software ensure that the analysis and management of medication therapy is restricted to pharmacists, whereas further tasks, such as entry of patient data, can also be performed by pharmacist technicians.

### Patient recruitment

Patients are provided consent materials and information on this research by pharmacy staff prior to initiation of the software-assisted MR service. Signed written consent forms remain at the pharmacies. Additionally, consent status is electronically documented by pharmacy staff in the software.

After completion of the MR process, pseudonymized datasheets of patients with electronic documentation of written consent are made available to the research group at the University of Muenster for patient selection and subsequent evaluation (see “data flow and protection” for details).

### Inclusion criteria

Patients are included in this study if they meet the following criteria:

- ≥ 65 years of age,
- ≥ 5 active pharmaceutical substances with systemic effects in long-term use (combinations in phytotherapeutic and homeopathic preparations are counted as single substance),
- written informed consent.

The inclusion of patients is limited to a maximum of 22 patients per pharmacy corresponding to 15% of the calculated sample size (*n* = 148). This limit was discussed in the research group and judged appropriate to increase external validity by limiting the impact of a single site on study results without eliminating too much data. In case of exceeding this limit at the end of recruitment period, study participants are randomly selected using a computer-generated list of random numbers. In addition, for patients with more than one completed MR during the recruitment period, only the first analysis is enclosed.

### Data collection in the MR software

**Fig. 1:**
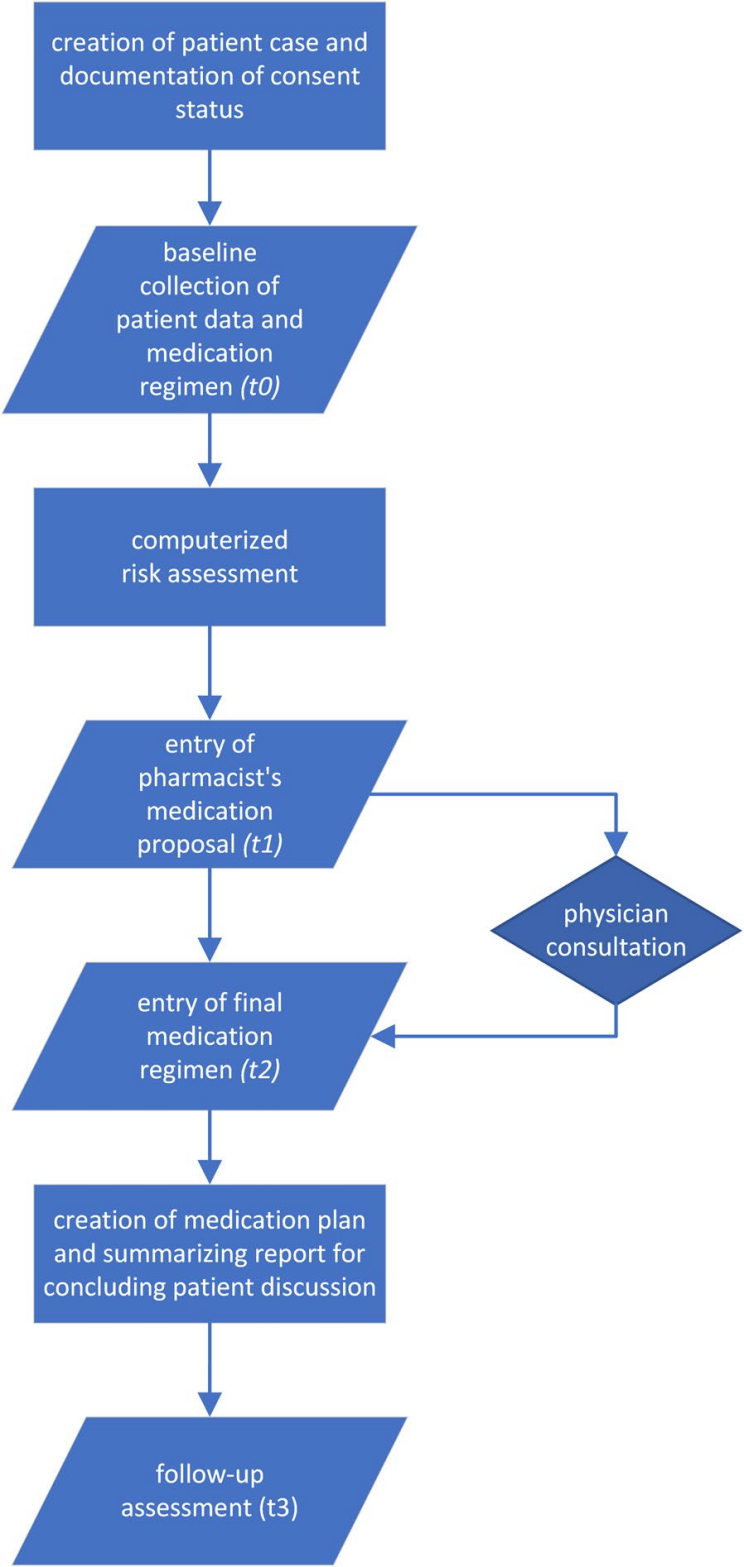
Flowchart of the medication review process in the software

This study is based on MR software-derived routine documentation. The following information is chronologically recorded in the software by community pharmacy staff over the course of the MR at four points in time (see Fig. 1):

- *t*_0_: baseline collection of patient data and medication regimen.
- *t*_1_: pharmacist’s medication proposal.
- *t*_2_: final medication regimen.
- *t*_3_: follow-up patient data.

Table 1 lists recorded data analyzed in this study.

**Table 1:** Collected data (upon availability) and corresponding times of documentation

Parameter	*t*_0_ Baseline	*t*_1_ Pharmacist’s medication proposal	*t*_2_ Final medication regimen	*t*_3_ Follow-up
***Patient data***				
Age (in years)	X			
Gender	X			
Weight and height	X			
Diagnosis (ICD-10-GM coded)	X			
Drug allergies	X			
Cytochrome-/drug-transporter status	X			
Laboratory values and vital signs (1)	X			
Consumption of tobacco, alcohol, food (e.g., grapefruit)	X			
Additional treatment-related information (free-text)	X			
Current symptoms	X			X
Medication-related quality of life	X			X
Pain (VAS 0–10)	X			X
Medication review-related goals	X			X
Adherence	X			X
Perception of the service				X
***Medication data for each drug/supplement***				
Medication (2)	X	X	X	
Directions, status (long-term, PRN, self-medication)	X	X	X	
Start of administration (date)	X			
Drug-related problems	X	X	X	

(1) Calcium, potassium, magnesium, sodium, serum creatinine, glucose, HbA1c, blood pressure, heart rate, respiratory rate, skin color for eGFR calculation)

(2) Including brand name, active ingredient, strength, dosage form, dosage regimen, national drug identification number, ATC-code

ICD-10-GM: International Statistical Classification of Diseases and Related Health Problems, 10. Version, German Modification; HbA1c: hemoglobin A1c; VAS: Visual Analogue Scale; ATC: Anatomical Therapeutic Chemical Classification; PRN: Pro re nata

In the following, the procedure and framework of data collection is outlined.

Baseline data collection (*t*_0_): Baseline data collection and entry is typically performed in the pharmacy in the context of a brown-bag review in a structured patient interview, taking into account other data sources, such as medication plans/lists, the pharmacy-internal medication history, and discharge letters. The patient’s medication regimen, clinical information, and first DRP identified during the interview (e.g., non-adherence or inappropriate storage) are recorded into the MR software by pharmacy staff. A software-integrated questionnaire comprising patient-reported outcome measures (PROM) is used for a standardized collection of patient perceptions and needs (e.g., symptoms, MR-related goals, adherence). The questionnaire can either be electronically filled by pharmacy staff during the patient interview or printed and self-completed in paper-based format. In case of paper-based completion of the form, data must be transferred manually into the software by pharmacy staff.

Computerized risk assessment and pharmacist’s medication proposal (*t*_1_): The CDSS performs a comprehensive risk assessment in six domains based on recorded clinical information and data from pharmaceutical databases:

- contraindication (due to age, gender, comorbidities, symptoms, lab values, drug allergies),
- indication (indication without drug; drug without indication),
- interactions (drug–drug; drug–food/alcohol/tobacco),
- potential side-effects (based on symptoms, diagnoses, lab values),
- adherence, administration (drugs/dosage forms with known risk for non-adherence and administration problems).

**Fig. 2:**
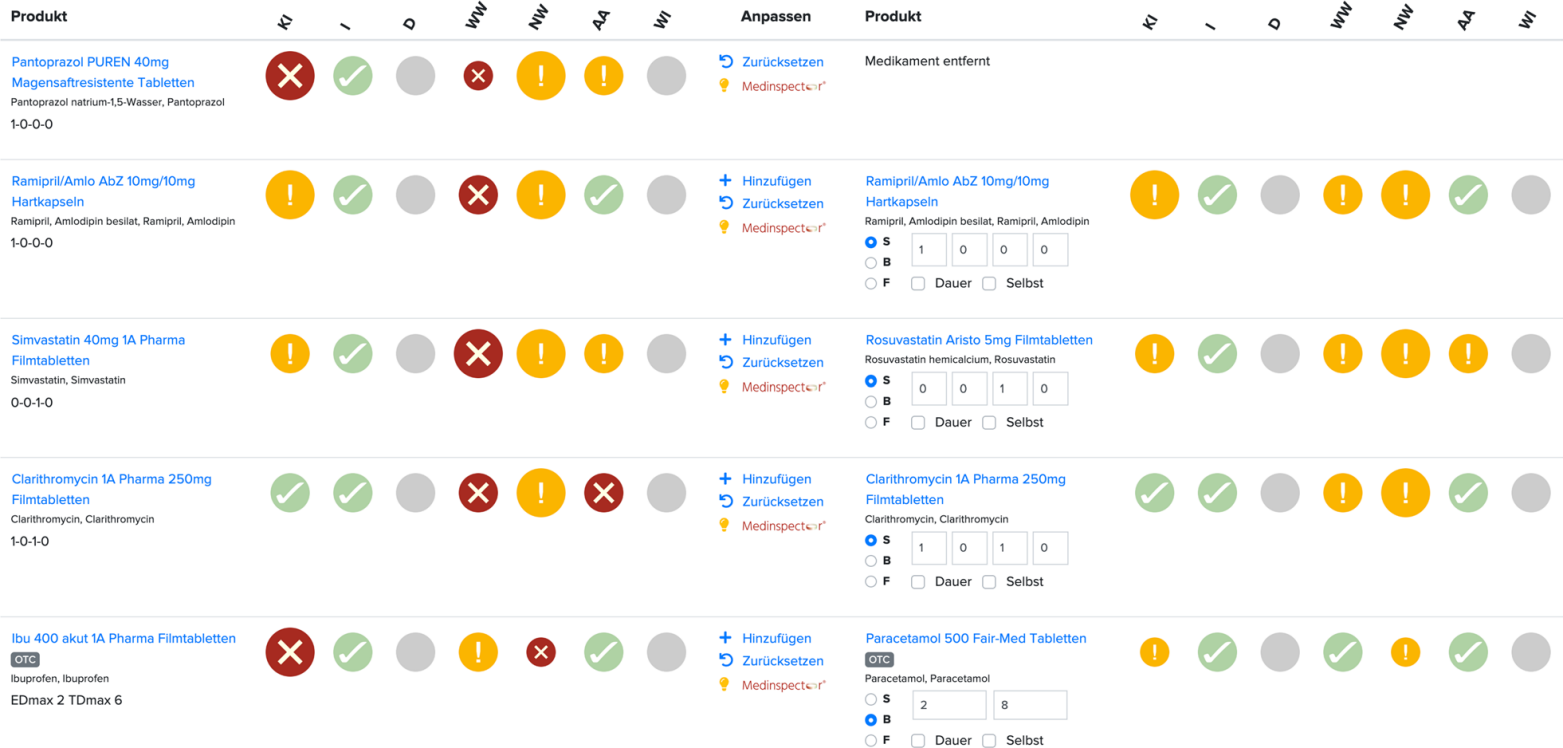
Overview of computerized risk assessment with before–after comparison of baseline medication (left) and a preliminary medication regimen (right). *Translation/Abbreviations* Produkt: product; KI: contraindication; I: indication; D: dosing (under development); WW: interactions; NW: side-effects; A/A: adherence/administration; WI: cost-effectiveness (under development); Anpassen: adjust; Hinzufügen: add; Zurücksetzen: reset; Dauer(medikation): long-term (medication); Selbst(medikation): self(-medication); Medikament entfernt: drug ceased

The computerized medication check includes information on nature and, in some cases, management of risks for each drug in a patient’s regimen. Based on the computerized assessment and patient preferences, pharmacists develop solutions for identified DRP by ceasing, starting, or changing drugs or their dosing scheme in the software. In this way, a preliminary medication regimen is recorded for a de novo risk examination. A visual comparison of risks before and after adjustment of medication allows an easy evaluation of effects of performed adjustment (see Fig. 2). Thus, multiple adjustments can be tested in order to develop a medication proposal. Subsequently, pharmacists document reasons for each adjustment in comparison to baseline medication. A customizable document generated on recorded data can be used to inform the prescribing physician about proposed adjustments. Final decisions about medication therapy remain at the discretion of the prescribing doctor.

Entry of the final medication regimen (*t*_2_): Pharmacists record the final medication, which is determined by the physician with the exception of self-medication. As a result, the German Federal Medication Plan along with a report summarizing the results of the MR can be created with the software to be provided to the patient during a subsequent concluding discussion of planned measures.

Follow-up (*t*_3_): As final step of the software-led MR process, an optional follow-up can be performed allowing an evaluation of effects of adopted measures and an initiation of further interventions if necessary. Therefore, the questionnaire applied during *t*_0_ and extended by an additional PROM can be used again for an assessment of patient perceptions and needs. Due to the non-interventional character of the study, it depends on clinical practice if or when follow-up is conducted.

### Data flow and protection

**Fig. 3:**
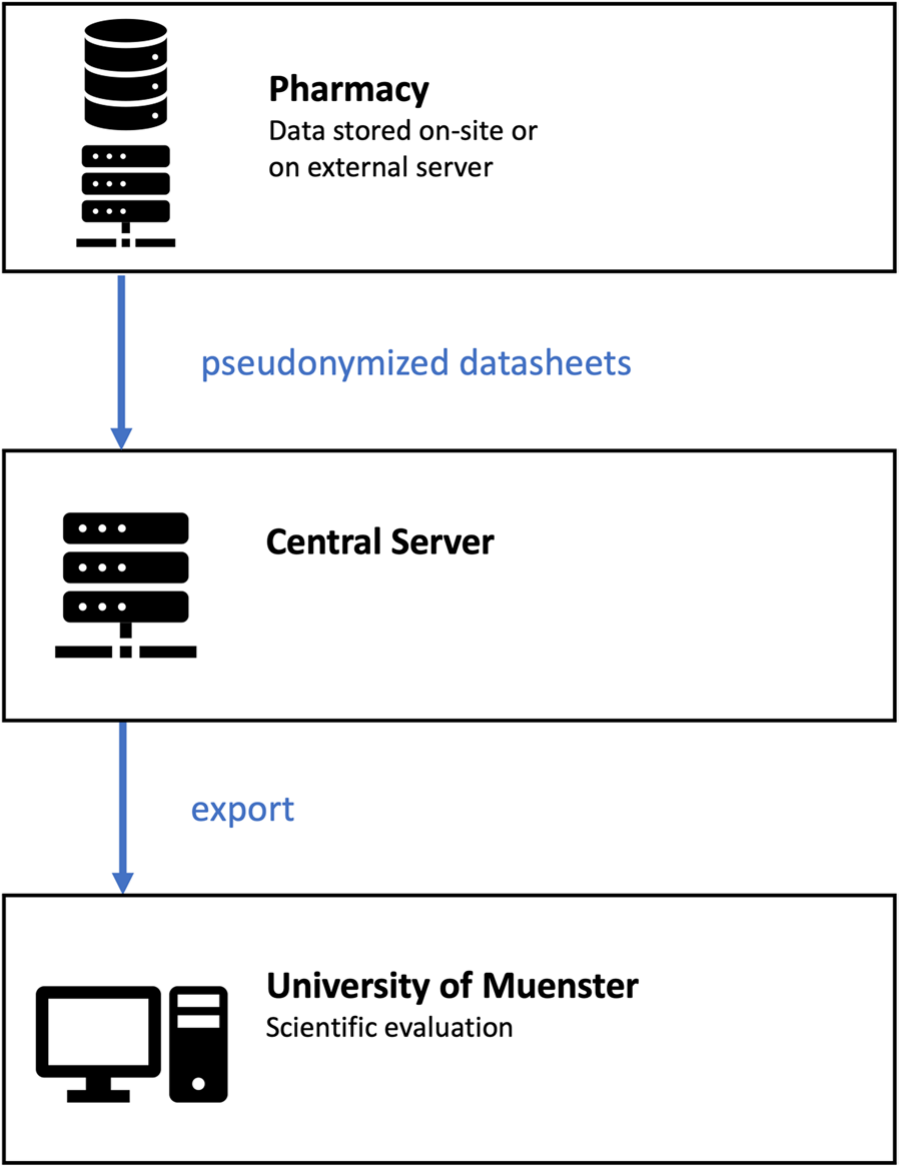
Data flow

Data recorded in the MR software of community pharmacies are either stored on local servers on-site or on external servers rented by the software provider. In addition, data of completed cases, for which patient consent for participation in this research were electronically documented, are automatically transferred to a central server. Data are transmitted in pseudonymized form and TLS (Transport Layer Security) encrypted. The secured central server is located in Germany and rented by the software provider. A member of the project group regularly accesses the central server via login-protected web-interface and exports a CSV (comma-separated values) file with datasheets of potential participants. Raw data are imported into Microsoft Excel and SPSS Statistics (IBM Corp., Armonk, USA) for the selection of patients based on inclusion criteria and subsequent statistical analysis. Figure 3 shows a schematic visualization of the study’s data flow.

All data transmitted for the purpose of scientific evaluation are pseudonymized automatically prior to leaving the pharmacies. Since we intend to consider only the first patient case for analysis, it is necessary to identify datasheets of patients with more than one completed MR. Consequently, a patient identifier (64-digit code) replacing the patient’s name is created by a secured hash algorithm (SHA-256). The generated code is based on the first and last name of a patient and thus in principle unique. Vice versa, it is impossible to draw conclusion from the generated code to the original name. Likewise, a pharmacy-ID-code is electronically created and added to each datasheet, thus, enabling the previous described limitation of included patients per pharmacy.

### Primary outcome

The primary outcome of this study is the appropriateness of medication which is assessed using the Medication Appropriateness Index (MAI), a judgement-based, validated instrument to measure inappropriate prescribing [23, 24]. Two research pharmacists with extensive experience in reviewing medication (IW, SM) rate the patient’s baseline medication (*t*_0_), the pharmacist’s medication proposal (*t*_1_) and the final medication regimen (*t*_2_) by applying the MAI in a slightly modified version. Raters follow operational instructions provided by the inventor of the instrument. Differences in MAI scores between *t*_0_ and *t*_2_ are used for primary outcome evaluation.

The original MAI consists of ten criteria which measure different characteristics of inappropriate pharmacotherapy and are assessed for each drug in a patient’s regimen. Each criterion is rated either appropriate, marginally appropriate, or inappropriate. A weighted score ranging from “1” to “3” is applied to criteria rated as inappropriate [25]. Subsequently, scores are added to a total MAI score per patient. Table 2 shows the MAI criteria and its corresponding scoring weights for the original and adapted version.

**Table 2:** The criteria of the MAI and its original and adapted weightings

Criterion	Original weight [25]	Adapted weight
1. Is there an indication for the drug?	***3***	***1***
2. Is the drug effective for the condition?	3	3
3. Is the dosage correct?	2	2
4. Are the directions correct?	2	2
5. Are the directions practical?	1	1
6. Are there clinically significant drug–drug interactions?	2	2
7. Are there clinically significant drug–disease/condition interactions?	2	2
8. Is there unnecessary duplication with other drug(s)?	1	1
9. Is the duration of therapy acceptable?	1	1
10. Is the drug the least expensive alternative compared to others of equal utility?	***1***	***–***

Differences between original and adapted weightings are highlighted in bold italics

We use an adaptation of the MAI specifically designed for the German healthcare system, which was recently used in a controlled trial and differs from the original version in two aspects [26, 27]:

- Despite being an unequivocally important criterion, “missing indication” is weighted with a score of “1” instead of “3” since complete and/or correct information on diagnoses are frequently not available in community pharmacies. Although the software used in this study facilitates the exchange of information between healthcare providers, we assume that data on diagnoses will still be missing in pharmacies to an extent that justifies this devaluation.
- An assessment of “cost-effectiveness” is excluded as non-transparent drug prices caused by discount agreements between statutory health insurances and pharmaceutical companies rule out an assessment of this criterion in Germany.

### Secondary outcomes

See Table 3 for a list of outcomes and instruments used in this study.

**Table 3:** Primary and secondary outcomes

Outcome parameter	Instrument	Outcome assessment
*Primary outcome*		
Appropriateness of medication	Medication Appropriateness Index (MAI), adapted	Change in MAI sum score **(*t***_**2**_ − ***t***_**0**_**)**
*Secondary outcomes*		
Appropriateness of medication	Medication Appropriateness Index (MAI), adapted	Change in MAI sum score **(*t***_**1**_ **−** ***t***_**0**_**)**
Medication underuse	Assessment of Underutilization of Medication Index (AOU)	Change in number of conditions with omitted drugs (*t*_2_ − *t*_0;_ *t*_1_ − *t*_0_)
Number of drugs in long-term use	Self-developed	Change in number of long-term drugs (*t*_2_ − *t*_0_; *t*_1_ − *t*_0_)
Exposition towards anticholinergic/sedative drugs	Drug Burden Index (DBI)	Change in DBI (*t*_2_ − *t*_0;_ *t*_1_ − *t*_0_)
Implementation of pharmacist-proposed adjustments in prescribed medication	Self-developed	Proportions of proposals (a) implemented, (b) partially implemented, (c) not implemented by physicians
Medication-related quality of life (PRO)	Seidling et al. [28]	Change in medication-related impairment after grouping response options “no”/“little” and “moderate”/“high”/“very high” into a dichotomous outcome (*t*_3_–*t*_0_)
Adherence (PRO)	Self-Rating Scale Item (SRSI), adapted	Change in adherence after grouping response options “very poor”/“poor” and “moderate”/“good”/“very good” into a dichotomous outcome (*t*_3_ − *t*_0_)
Symptom burden (PRO)	Self-developed	Change in number of (a) total symptoms, (b) symptoms rated “moderate”/“severe” (*t*_3_ − *t*_0_)
Fulfillment of medication review-related goals (PRO)	Self-developed	Proportions of goals fulfilled (a) “not at all”, (b) “partially”, (c) “completely” (at *t*_3_)
Perception of the MR service (PRO)	Self-developed	Distribution of answers on 5-point Likert scale (five items)

PRO: patient-reported outcome

#### Medication-related outcomes

Differences in the MAI between *t*_0_ and *t*_1_ are analyzed as a secondary outcome variable. Further process measures are changes between *t*_0_ and *t*_2_ as well as *t*_0_ and *t*_1_ in number of long-term drugs, medication underuse (Assessment of Underutilization of Medication Index, AOU), and exposition towards anticholinergic/sedative drugs (Drug Burden Index, DBI) as well as implementation rate of pharmacist-proposed adjustments in prescribed medication [29, 30].

#### Patient-reported outcomes (PRO)

Data for the analysis of PRO are derived from questionnaires applied at baseline (*t*_0_) and follow-up (*t*_3_). Questionnaires were developed at the University of Muenster in cooperation with the software distributor with the aim to be equally suitable to collect outcome data for this research and also clinically relevant information in daily practice. Due to a partial lack of easy and quick to use alternatives fitting the needs of pharmacists and their patients in routine care, the questionnaire includes self-developed PROM.

A single item question created by Seidling and colleagues is used at *t*_0_ and *t*_3_ to measure *medication-related quality of life* [28].

*Adherence* is assessed at *t*_0_ and *t*_3_ with an adaptation of the validated Self-Rating Scale Item (SRSI) [31, 32]. We chose this instrument since it covers all aspects of adherence problems, namely intentional and non-intentional non-adherence as well as related over-, under-, and misuse of drugs. Patients rate their capability to use their drugs in accordance with instructions provided by healthcare professionals over the past 4 weeks. Compared to the original version, we removed the response option “excellent” resulting in a modified instrument with five categories ranging from “very poor” to “very good”. This adaptation was made since cognitive pretests revealed a rejection of this option in favor of the second highest ranked response (“very good”), even though the ability to follow the instructions was considered optimal by the test candidates with no room for improvements left. This pattern in responses is most likely attributed to “very good” being the maximum achievable grade in the German schooling system. Furthermore, we translated the instrument into German language in accordance with the ISPOR guidelines for the translation and cultural adaptation of PROM [33].

To measure patient-reported *symptom burden*, we designed a rating-scale comprising 18 symptoms/symptom categories commonly linked with adverse drug reactions in elderly patients. Symptoms were selected on the basis of published research and the evaluation of MR from the medication therapy safety training program “Apo-AMTS” [34, 35]. At *t*_0_ and *t*_3_, patients rate the extent of symptoms experienced over the past 4 weeks on a four-point scale (“non-existing”, “mild”, “moderate”, “severe”).

The degree of *fulfillment of MR-related goals,* set by the patient at *t*_0_, is measured at *t*_3_ on a three-point scale (“not at all”, “partial”, “complete”). The self-developed instrument is composed of eight individualizable categories of patient goals, which were frequently reported for MR in previously published literature and the “Apo-AMTS” program [36].

At *t*_3_, a self-designed instrument with five items is used to capture patient’s perception of the MR service. Patients rate perceived changes and benefits attributed to the service on a 5-point Likert scale in the following dimensions of medication therapy: knowledge on drugs, confidence in pharmacological treatment, handling of drugs, shared decision-making, health benefits.

### Statistical analyses

#### Sample size calculation

Based on the results obtained in a previous study, an effect size of *d*_Cohen_ = − 0.25 was assumed for the primary outcome [37]. With a lack of an established definition of a minimal clinically important difference in the MAI, we consider this effect to be clinically relevant and feasible [38]. In order to detect this effect with a two-sided Wilcoxon signed-rank test at 80% power and a significance level of 5%, 148 evaluable patients are needed. The power analysis was performed with G*Power version 3.1.9.2 [39].

#### Baseline characteristics

Baseline characteristics will be analyzed descriptively with calculation of parameters of central tendency and dispersion for quantitative variables and number and proportions for qualitative variables.

#### Outcome evaluation

We will perform descriptive and interferential analyses of the primary and secondary outcomes.

Change in MAI between *t*_0_ and *t*_2_ will be tested for significance by using a two-sided Wilcoxon signed-rank test (*α* = 0.05). The expected change in MAI is highly dependent on pre-interventional medication appropriateness. In a recent trial, a low MAI at baseline did not provide enough scope for improvement [20]. A baseline MAI of ≥ 24 points was identified by Rose et al. as a potential cut-off value for the selection and prioritization of patients with significantly higher expected benefit from MR [40]. Hence, we will perform subgroup analyses for patients with comparably low and high medication appropriateness at baseline, defined as a MAI < 24 points and ≥ 24 points, respectively. Kappa statistics will be used to assess interrater reliability of MAI evaluation. The analysis of secondary outcomes will follow a prespecified statistical analysis plan.

## Discussion

Geriatric polypharmacy has become a major public health concern worldwide [1]. The current “Aktionsplan Arzneimitteltherapiesicherheit 2021–2024” (action plan medication therapy safety) by the German Federal Ministry of Health recommends the use of digital technologies to enhance medication therapy safety [41]. This approach is realized with the software Medinspector^®^, a novel CDSS for structured MR in community pharmacies. In our opinion, the software concept is innovative in two ways: firstly, drug assessment is not limited to the initial medication, as considered adjustments in a regimen are assessed dynamically. This approach takes into account that every adjustment in medication may trigger new problems, thus, allowing software-users a careful consideration of potential benefits and risks of different treatment options. Secondly, users may request case-specific support from pharmacist-experts trained in MR and employed at the software provider. To date, MR has not been implemented in Germany on a broad basis and a significant proportion of pharmacists is expected to have limited experience in delivering this service [42]. We believe getting support from trained experts in case of uncertainty will encourage pharmacists to initiate therapy modifications more often.

The observational OPtiMed study is designed to determine the effects of utilizing this software in MR on medication-related and patient-reported outcomes in elderly community pharmacy patients with polypharmacy. We assume that the MR service will improve the quality of pharmacotherapy, thereby resulting in improvements of PRO, such as health-complaints and medication-related quality of life.

We chose the MAI to be the primary outcome in this study as it is a valid and reliable instrument that measures multiple outcomes recommended in a recently developed core outcome set (COS) for MR studies, namely overuse, potentially inappropriate drugs, and clinically significant drug–drug interactions. To complement the MAI, we will investigate medication underuse in line with the recommendation of the COS [43].

In most previous studies, MAI assessment was limited to the medication utilized at baseline and after completion of the MR [11, 12]. We decided to additionally evaluate MAI scores and further process outcomes for the medication regimen proposed by pharmacists. Insight into the quality of proposed medication will help us to determine the isolated effects of using the software for medication optimization, irrespective of a subsequent implementation of proposed adjustments by the physicians.

It was of utmost importance for us to include patients’ perspective and experience in this research. As part of the software development process, we designed a questionnaire for PRO data collection combining self-developed PROM with PROM from the literature. From the beginning, the questionnaire was intended to become an integral and permanent component of the MR software. Hence, we aimed to design and select only instruments perceived as clinically useful by pharmacists and their patients in daily practice: more specifically, quick and easy to use instruments that increase pharmacists’ ability to address patients’ needs in the context of MR.

Results from a trial conducted by Verdoorn and colleagues suggest a more patient-centered approach in MR [36]. With software-implementation of carefully selected PROM with specific focus on patient goals, preferences, and health-related complaints, we intend to foster patient-oriented care. For example, patients are encouraged to set personal goals during MR, allowing pharmacists and physicians to prioritize problems and derived interventions.

Some limitations pertaining to the collection of PRO data need to be addressed. First, we did validate self-developed PROM; however, all instruments were thoroughly designed and pretested. Second, the questionnaire is used by pharmacists and patients in daily practice and not in standardized manner by a briefed member of the research team. It must therefore be assumed that items are—unintentionally or intentionally—not always used as expected, although we particularly focused on providing easy to use instruments with low risk of misinterpretation. Third, the study is an uncontrolled before–after study. Thus, in particular, patient-related outcomes could be affected by some other influential events between baseline and follow-up, making it difficult to attribute observed changes solely to the MR. Moreover, this investigation lacks a follow-up of medication changes after completion of the MR. An earlier study demonstrated that changes in medication and further measures planned during MR were frequently implemented with delay [28]. Consequently, our research will likely not capture all effects of the intervention, e.g., in case the clinical situation of a patient requires a stepwise implementation rather than a change of multiple drugs at once. Another constraint of this study is a possible reduction in external validity due to the involvement of pharmacists participating in a MR teaching program. Participants’ performance may differ from “standard” software-users, as they have received comprehensive education on how to conduct MR but regularly lack routine in providing this service. However, from our knowledge, a significant proportion of MR performed in Germany at present are executed in the context of such training programs. Hence, the involvement of this group may reflect current routine care in Germany.

**Trial status**: At the time of submission of this manuscript, recruitment is ongoing. Recruitment started in February 2022 and is expected to be completed by January 2023.

## Data Availability

Not applicable.

## Abbreviations

AOU: Assessment of Underutilization of Medication Index
CDSS: Clinical decision support system(s)
DBI: Drug Burden Index
DRP: Drug-related problem(s)
MAI: Medication Appropriateness Index
MR: Medication review(s)
PRO: Patient-reported outcome(s)
PROM: Patient-reported outcome measure(s)
SRSI: Self-Rating Scale Item
WHO: World Health Organization
